# Application of Genetic Algorithms in Nonlinear Heat Conduction Problems

**DOI:** 10.1155/2014/451274

**Published:** 2014-02-17

**Authors:** Muhammad Bilal Kadri, Waqar A. Khan

**Affiliations:** ^1^Department of Electronics and Power Engineering, PN Engineering College, National University of Sciences and Technology, PNS Jauhar, Karachi 75350, Pakistan; ^2^Department of Engineering Sciences, PN Engineering College, National University of Sciences and Technology, PNS Jauhar, Karachi 75350, Pakistan

## Abstract

Genetic algorithms are employed to optimize dimensionless temperature in nonlinear heat conduction problems. Three common geometries are selected for the analysis and the concept of minimum entropy generation is used to determine the optimum temperatures under the same constraints. The thermal conductivity is assumed to vary linearly with temperature while internal heat generation is assumed to be uniform. The dimensionless governing equations are obtained for each selected geometry and the dimensionless temperature distributions are obtained using MATLAB. It is observed that GA gives the minimum dimensionless temperature in each selected geometry.

## 1. Introduction

In many engineering and biological systems, conduction heat transfer plays a vital role. The basic information on conduction heat transfer can be found in any text book [1–3]. Many researchers, including [4–16], have investigated analytically/numerically the problems of steady/unsteady conduction heat transfer in different geometries with different boundary conditions.

Davalos and Rubinsky [17] incorporated, perhaps for the first time, the ideas from the fields of evolution and genetics in a new approach to solve heat transfer problems. They also demonstrated the feasibility of this new approach to solving problems in physics. Later on, Rubinsky and Davalos [18] solved the conduction heat transfer equation by using genetic algorithms. But their analysis was limited to very coarse meshes. Tsourkas and Rubinsky [19] not only resolved this problem but also extended their analysis to fine meshes. They also studied the effect of different parameters and operators on the accuracy of the results. Tsourkas and Rubinsky [20] developed genetic algorithms for implementation on parallel computers to solve heat conduction problems. Their algorithm involved a novel local search operator that greatly improves its accuracy. Gosselin et al. [21] presented a comprehensive review of the application of genetic algorithms in heat transfer problems. They presented a number of papers in which heat conduction was optimized. They identified three main types of heat transfer problems related to thermal systems, inverse heat transfer problems and development of heat transfer correlations. Felczak and Wicek [22] implemented genetic algorithm for electronic devices placement optimization. They assumed that nine electronic devices can be positioned randomly on a substrate surface and created a 2-level optimization algorithm. They optimized the positions of nine electronic devices with respect to thermal criteria and wiring length.

Genetic Algorithm is based on the Darwinian principle of evolution and it follows the principle of “survival of the fittest.” Genetic algorithms, proposed by Holland [23] and enhanced by others [24, 25], have shown exponential growth in various fields of engineering and technology ranging from control theory, signal processing, antenna design, biomedical signal processing, and so forth [26–28]. Genetic algorithms have been successfully applied in the field of heat transfer since mid-1990s [29, 30]. Complex optimization involving nonlinear constraints can be easily solved using genetic algorithms [31]. Conventional deterministic optimization techniques have immense computational requirement and can only be applied when the objective function is differentiable and continuous [32]. Conventional optimization schemes have a drawback of converging in local minima; hence, the solution is compromised [33]. Deterministic optimization schemes often fail when the objective function is multiextremal [34, 35].

## 2. Genetic Algorithm

Genetic algorithms have been successfully applied in the field of heat transfer since mid-1990s. Complex optimization involving nonlinear constraints can be easily solved using genetic algorithms. Optimization problems that cannot be solved because of the extremely massive computational requirements can be easily solved using genetic algorithms. Genetic algorithms are based on heuristic approach and searches for the best individuals. A generation consists of randomly generated population. The members of the population are tested for fitness. The most suitable candidates are allowed to reproduce the new generation. The next generation consists of the best individuals from the previous generation and their offspring. The chromosomes of each member are also mutated to generate a more suitable candidate in the next generation.

The fitness function (the function to be minimized) is the entropy generation in all the cases. The simulation is developed in MATLAB. The fitness scores, returned by the fitness function, are ranked on the basis of their scores. The rank of the fittest individual is set to “1” and the next fittest is assigned a rank of “2” and so on. Rank fitness scaling removes the effect of raw scores. “Stochastic uniform” scaling function is used for selection of parents in the next generation. The parents are selected based on the rank as determined earlier. Stochastic uniform algorithm lays out a line in which each parent corresponds to a section of the line of length proportional to its expectation. The algorithm moves along the line in steps of equal size, one step for each parent. At each step, the algorithm allocates a parent from the section it lands on. The first step is a uniform random number less than the step size. Reproduction algorithm determines how the offspring are generated. The elite count is set to “2” which guarantees that a maximum of two individuals will survive in the next population. Crossover generates member of the new generation by combining genes from two individuals from the previous generation. The crossover process used is “scattered” which first creates a random binary vector. A “1” in the random binary vector is replaced by genes from the first parent and a “0” in the random binary vector is replaced by a gene from the second individual. Crossover fraction is a factor which determines how many individuals in the next generation will be generated by mixing genes from two different chromosomes. The crossover fraction has a value of 0.8. The remaining members of the generation will be generated by the mutation process. In the mutation process one or two genes are inverted in order to generate a better diversity in the population. The mutation function used in our experiment is “Gaussian.” Gaussian adds a random number to each individual; the random number is derived from a Gaussian distribution having zero mean and a standard deviation of 1. In all the experiments the size of the population in each generation was set to 10. Total generations were set to 10. The optimized values from the genetic algorithm for the three selected geometries are shown in Table 1.

## 3. Mathematical Analysis

Consider one-dimensional steady conduction in a plane wall of thickness *L*, a hollow cylinder of inside radius *r*_1_, outside radius *r*_2_, and length *L* or a hollow sphere of inside radius *r*_1_ and outside radius *r*_2_ with temperature dependent thermal conductivity *k* = *k*_1_[1 + *β*(*T* − *T*_1_)], where *β*(*K* ^−1^) is a measure of the thermal conductivity variation with temperature and *k*_1_ is the thermal conductivity at temperature *T*_1_. Each geometry is experiencing a uniform volumetric heat generation at the rate $\dot{q}$. The temperature on the left face of the wall or at the inner radius is *T*_1_ while the temperature on the right face or at the outer radius is *T*_2_, where *T*_2_ > *T*_1_. The dimensionless governing equations for a plane wall, hollow cylinder, and hollow sphere with temperature dependent thermal conductivity and uniform volumetric heat generation can be written as

(1) ddX{[1+a(θ−1)]dθdX}+Q˙=0  (Plane wall),1RddR{[1+a(θ−1)]RdθdR}+Q˙=0  (Hollow cylinder),1R2ddR{[1+a(θ−1)]R2dθdR}+Q˙=0  (Hollow sphere),

with *boundary conditions*

(2) θ(0)=1, θ(1)=θ∗  (Plane wall),θ(1)=1, θ(R∗)=θ∗  (Hollow  cylinder  and  sphere),

where

(3) θ=TT1, θ∗=T2T1, a=βT,X=xL  (Plane wall),R=rr1, R∗=r2r1  (Hollow cylinder or sphere),Q˙=q˙L2kT1  (Plane wall),Q˙=q˙r12kT1  (Hollow cylinder or sphere).

The governing equations given in (1) are nonlinear due to temperature dependent thermal conductivity and do not have exact analytical solutions. In this study, numerical solution is obtained by using MATLAB. The equations were solved using MATLAB built-in function “bvp4c.” In order to use this function (1) was transformed into a system of equations. The boundary conditions were specified in each case. The mesh size (used to solve the boundary value problem) was set to 10, that is, 10 equidistant points between initial and final values of temperatures.

### 3.1. Entropy Generation Rates

Once the dimensionless temperature distribution is known, the entropy generation rate for the selected geometries can be calculated using the expressions for the volumetric entropy generation rates given by Bejan [36]. In dimensionless form, the entropy generation rates ${\dot{S}}_{\text{gen}}$ for the selected geometries can be written as

(4) S˙genLkA=Sgen∗=∫01[1+a(θ−1)](1θdθdX)2dX(Plane  wall),S˙gen2πk1L=Sgen∗=∫1R∗[1+a(θ−1)]R(1θdθdX)2dR         (Hollow  cylinder),S˙gen4πk1r1=Sgen∗=∫1R∗[1+a(θ−1)]R2(1θdθdX)2dR           (Hollow  sphere).

These expressions were also evaluated numerically using MATLAB. The solution of the dimensionless temperature, that is, *θ*(*X*) or *θ*(*R*) (depending upon the geometry) is obtained by utilizing “bvp4c”. A third order polynomial was approximated from the solution. The integral for the entropy generation rates in (4) for each of the three cases was solved by invoking MuPad facility from the MATLAB environment. The solution for the definite integral was found to be very accurate. The variation of entropy generation rate with respect to "a" and "Q" for three geometries are shown in Figures 3, 5 and 7 respectively.

**Figure 1:**
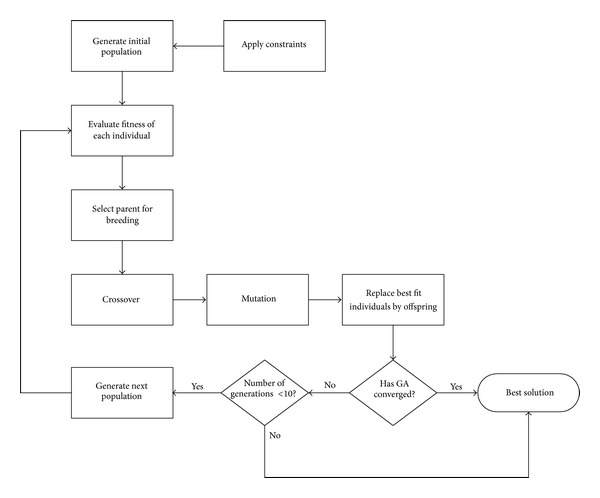
Flowchart for the optimization process.

The optimization process is shown in Figure 1. An initial population is generated which should lie within the constraints defined on the parameters. The constraints are applied on *a*,$\dot{Q}$, and θ*, respectively. Each member of the population is evaluated for fitness. The most suitable individuals (having the best fitness values) are allowed to breed. The breeding process (in a broad sense) consists of crossover and mutation; the bad individuals are replaced by the best offspring. The genetic algorithm checks for the convergence. If the algorithm converges then it terminates otherwise it generates a new set of population [37]. In our experiment the maximum number of generations are set to 10. If 10 generations is produced then the algorithm terminates giving the best value of ${\dot{S}}_{\text{gen}}$ (objective function) in the 10th generation.

## 4. Theoretical Foundation of Genetic Algorithms

Genetic algorithm is used for optimizing functions. Let the objective function to be minimized be denoted by $f(x)={\dot{S}}_{\text{gen}}$, where *x* = {*x*_*t*_ | *t* = 1,2,…., *N*_*k*_}. “*t*” denotes the parameter space of the optimization problem. *N*_*k*_ is the maximum number of parameters to be considered in the optimization process. “*x*_*t*_” can be continuous or discrete, real or imaginary [26]. The genetic algorithm transforms the parameter's values *x*_*t*_ into a symbolic representation “*d*” which is termed as a chromosome. Many genes combine to form a chromosome. The number of genes in a chromosome determines the quantization level of the parameters (used in optimization process). The chromosome “*d*” can be represented by

(5) $$\begin{array}{c} d=\left\{e_{j}\;|\;j=1,2,\ldots ,N_{dl}\right\}, \end{array}$$

where *N*_*dl*_ is the length of the chromosome. The encoding of a parameter value “*x*_*t*_” into “*d*” can be represented by

(6) $$\begin{array}{c} x_{t}=x_{t}^{\min}+2^{-N_{t-1}}\frac{x_{t}^{\max}-x_{t}^{\min}}{2^{N_{t}-N_{t-1}-1}}\sum_{j=N_{t-1}+1}^{N_{t}}e_{j}2^{j-1}, \end{array}$$

where *x*_*t*_^min⁡^ and *x*_*t*_^max⁡^ are the minimum and maximum values of the parameter *x*_*t*_. A generation *Z* consists of randomly generated population:

(7) $$\begin{array}{c} Z=\left\{z_{i}\;|\;i=1,2,3,\ldots ,R\right\}, \end{array}$$

where “*R*” is the maximum number of members in the population. The members of the population are tested for fitness. In our specific case the members are tested so that they lie within the bounds defined on the parameters. The filtered individuals are used to determine the cost (of the objective function). Production of new members in the next population consists of three major steps, namely, selection, crossover, and mutation. the most suitable candidates are selected as parents and are allowed to reproduce the new generation. The selectionoperation can be represented by *Z*_*S*_^*k*^ = *S*(*Z* ^*k*^) which produces a population of size “*R*.” Various selection schemes have been proposed such as ranking, roulette-wheel, and stochastic uniform methods [25]. The next generation consists of the best individuals from the previous generation and their offspring. Chromosomes from two parents are combined to generate an offspring; this process is termed as crossover process. Crossover can be denoted as the union over a composition of operators and is given by

(8) $$\begin{array}{c} C\left(Z_{S}^{k}\right)=\overset{R/2}{\underset{i=1}{⋃}}c\left[ty\left(Z_{S}^{k}\right),ty\left(Z_{S}^{k}\right)\right], \end{array}$$

where the operator “*ty*” chooses a random chromosome from *Z* and the operator “*c*” maps a pair of chromosomes *z*_1_ = {*e*_1*j*_ | *j* = 1,…, *N*_*dl*_} and *z*_2_ = {*e*_2*j*_ | *j* = 1,…, *N*_*dl*_} to another pair such that:

(9) $$\begin{array}{c} c\left(z_{1},z_{2}\right)=\{\begin{array}{cc} {\overset{-}{z}}_{1},{\overset{-}{z}}_{2} & \text{with}\;\text{probability}\;p_{\text{cross}}\\ z_{1},z_{2} & \text{with}\;\text{probability}\;1-p_{\text{cross}}, \end{array} \end{array}$$

where the newly produced offspring are given by

(10) $$\begin{array}{c} {\overset{-}{z}}_{1}=\left\{e_{11},e_{12},\ldots ,e_{1k},e_{2(k+1)},e_{2(k+2)},\ldots ,{e_{2}}_{N_{dl}}\right\},\\ {\overset{-}{z}}_{2}=\left\{e_{21},e_{22},\ldots ,e_{2k},e_{1(k+1)},e_{1(k+2)},\ldots ,{e_{1}}_{N_{dl}}\right\}. \end{array}$$

The chromosomes of each member are also mutated (the value of a randomly selected gene is inverted from 0 to 1 or 1 to 0) to generate a more suitable candidate in the next generation. The mutation process is given by *Z*_*M*_^*k*^ = *M*(*Z*_*C*_^*k*^) and mathematically it can be represented as

(11) $$\begin{array}{c} M\left(Z_{C}^{k}\right)=\overset{R}{\underset{i=1}{⋃}}m\left({\tilde{z}}_{i}^{k}\right). \end{array}$$

For a chromosome *z* = {*e*_*j*_ | *j* = 1,…, *N*_*dl*_}, *m*(*z*) = {*ψ*(*z*_*j*_) | *j* = 1,…, *N*_*dl*_}, and

(12) $$\begin{array}{c} \psi \left(z\right)=\{\begin{array}{cc} \tilde{z} & \text{with}\;\text{a}\;\text{probability}\;p_{\text{mut}}\\ z & \text{with}\;\text{a}\;\text{probability}\;1-p_{\text{mut}}. \end{array} \end{array}$$

## 5. Simulation Setup

The fitness function (the function to be minimized) is the entropy generation rate in all the cases. The simulation is developed in MATLAB. The fitness scores returned by the fitness function are ranked on the basis of their scores. The rank of the fittest individual is set to “1” and the next fittest is assigned a rank of “2” and so on. Rank fitness scaling removes the effect of raw scores. “Stochastic uniform” scaling function is used for selection of parents in the next generation. The parents are selected based on the rank as determined earlier. Stochastic uniform algorithm lays out a line in which each parent corresponds to a section of the line of length proportional to its expectation. The algorithm moves along the line in steps of equal size, one step for each parent. At each step, the algorithm allocates a parent from the section it lands on. The first step is a uniform random number less than the step size. Reproduction algorithm determines how the offspring are generated. The elite count is set to “2” which guarantees that a maximum of two individuals will survive in the next population. The crossover process used is “scattered” which first creates a random binary vector. A “1” in the random binary vector is replaced by genes from the first parent and a “0” in the random binary vector is replaced by a gene from the second individual. Crossover fraction is a factor which determines how many individuals in the next generation will be generated by mixing genes from two different chromosomes. The crossover fraction has a value of 0.8. The remaining members of the generation will be generated by the mutation process. The mutation process generates a better diversity in the population. The mutation function used in our experiment is “Gaussian.” Gaussian adds a random number to each individual; the random number is derived from a Gaussian distribution having zero mean and a standard deviation of 1. In all the experiments the size of the population in each generation was set to 10, that is, *N*_*dl*_ is set to 10. Total generations were set to 10. The optimized values from the genetic algorithm for plane wall, hollow cylinder, and hollow sphere are shown in Table 1.

## 6. Results and Discussion

The genetic optimization tool in the MATLAB environment was used to solve nonlinear heat conduction problem in three selected geometries. The most important task is to code the fitness function and to set all the constraints. The genetic algorithm calls the fitness function at each instant with random values of the parameters to be optimized. These random values are the members of the population. With each set of values, the boundary value problem is solved and the numerical solution is converted into a third degree polynomial. The polynomial is subsequently used (by invoking MUPAD from MATLAB) to determine the entropy generation rate. The entropy generation rate is determined for the complete population and the minimum value of the entropy generation rate is held and the remaining values are discarded.

**Figure 2:**
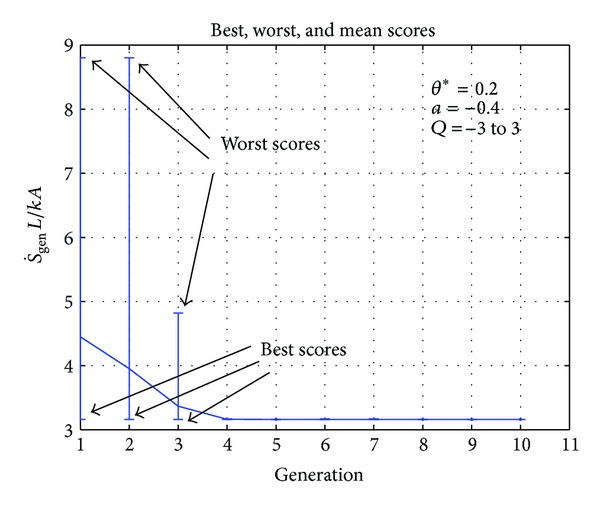
Best, worst, and mean values of fitness function (${\dot{S}}_{\text{gen}}$) for plane wall.

**Figure 3:**
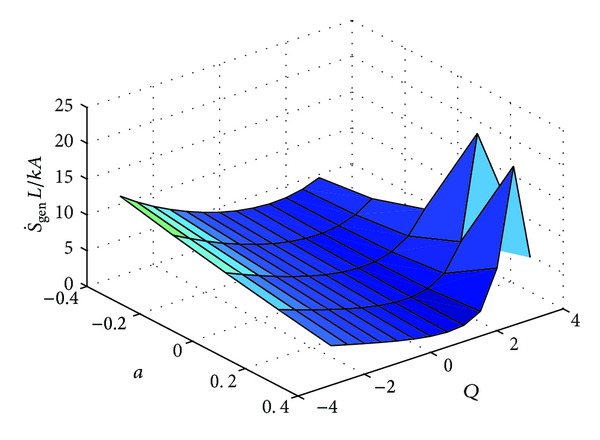
Variation of ${\dot{S}}_{\text{gen}}$ with respect to “*a*” and “*Q*.”

**Figure 4:**
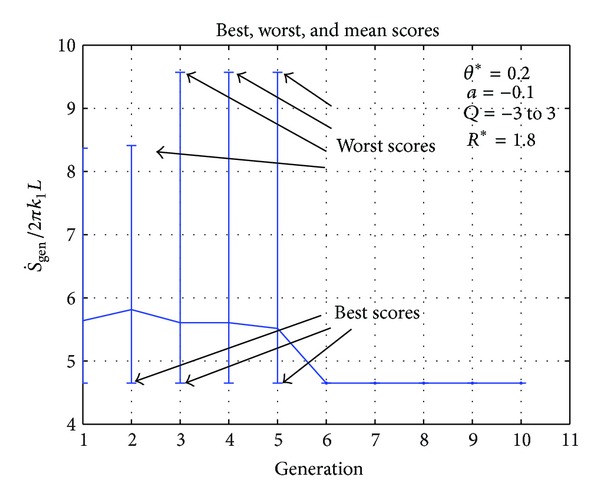
Best, worst, and mean values of fitness function (${\dot{S}}_{\text{gen}}$) for hollow cylinder.

**Figure 5:**
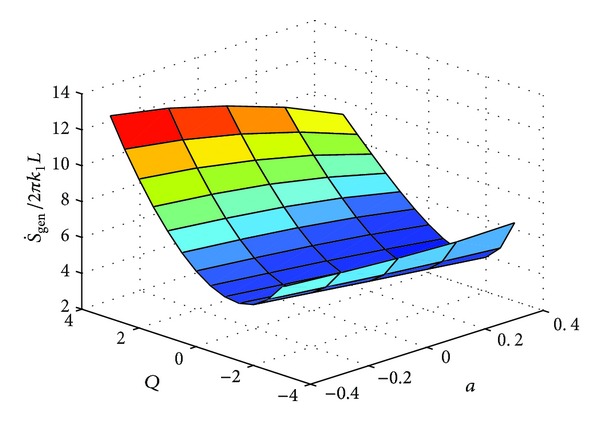
Variation of ${\dot{S}}_{\text{gen}}$ with respect to “*a*” and “*Q*.”

**Figure 6:**
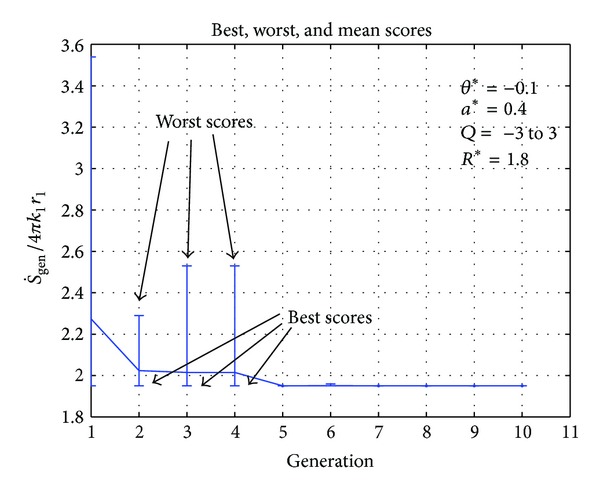
Best, worst, and mean values of fitness function (${\dot{S}}_{\text{gen}}$) for hollow sphere.

**Figure 7:**
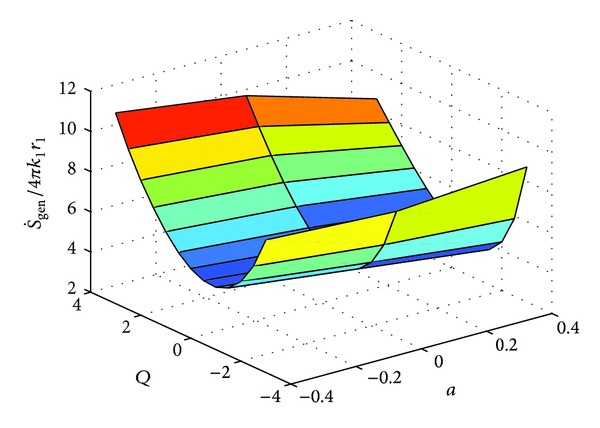
Variation of ${\dot{S}}_{\text{gen}}$ with respect to “*a*” and “*Q*.”

One case for each of the selected geometries that is, plane wall, hollow cylinder, and hollow sphere, is shown in Figures 2–7, respectively. In Figure 2, initially the fitness function value is above 4 and the worst score varies between 3 and 9 but when more members of the population are generated, the fitness value converges. It can be observed that after the fourth generation the fitness value does not change. In the case of hollow cylinder, the fitness values converge in the sixth generation. There is a lot of variation in the initial generations as can be seen in Figure 4 but the solution gradually converges. For the case of hollow sphere (Figure 3), the solution converges in the fifth generation. The vertical lines in all the figures represent the maxima and minima generated in each generation. In all the cases there are vertical lines for the initial generations but the vertical lines do not appear in the later generations. This clearly shows that only the best individuals are retained and they migrate from one generation to another.

## 7. Conclusions

Genetic algorithms are successfully employed to optimize the dimensionless temperature in the selected geometries. The concept of minimum entropy generation was used to obtain the minimum dimensionless temperature. It is found that, for the same constraints, the minimum dimensionless temperature and hence the minimum entropy generation rates are obtained in case of plane wall.

## Figures and Tables

**Table 1:** Comparison of entropy generation rates for the three selected geometries when $-3\leq \dot{Q}\leq 3\;\text{and}\;R^{*}=1.8$.

	*a*	θ*	${\dot{S}}_{\text{gen}}$	
			GA	Numerical
Plane wall	−0.4	0.2	3.16	3.12
	0		2.62	2.61
	0.4		2.07	2.09
	−0.4	0.4	0.957	0.95
	0		0.84	0.84
	0.4		0.723	0.72

Hollow cylinder	−0.1	0.2	4.65	4.73
	0		4.46	4.50
	0.1		4.28	4.28
	−0.1	0.4	1.47	1.48
	0		1.43	1.43
	0.1		1.38	1.39

Hollow sphere	−0.1	0.2	6.33	6.36
	0		6.07	6.06
	0.1		5.8	5.77
	−0.1	0.4	1.95	1.97
	0		1.89	1.91
	0.1		1.85	1.84

## Nomenclature

*a*: Thermal conductivity parameter, *βT*_1_, dimensionless
*A*: Area of plane wall normal to the direction of heat transfer, m^2^
*k*: Thermal conductivity, W/m·K
*k*_1_: Thermal conductivity at *T* = *T*_1_, W/m·K
*L*: Thickness of plane wall or the length of hollow cylinder, m
*r*: Radial coordinate, m
*r*_1_: Inside radius of hollow cylinder or hollow sphere, m
*r*_2_: Outside radius of hollow cylinder or hollow sphere, m
*R*: Dimensionless radial coordinate, *r*/*r*_1_
*R**: The ratio of outside to inside radius, *r*_2_/*r*_1_
${\dot{S}}_{\text{gen}}^{\prime \prime \prime }$: Entropy generation rate per unit volume, W/m^3^·K
${\dot{S}}_{\text{gen}}$: Entropy generation rate, W/K
*T*: Temperature, K
*x*: Distance, m
*X*: Dimensionless distance, *x*/*L*.

### Greek Symbols

*β:*: Thermal conductivity coefficient, K^−1^
*θ*: Dimensionless temperature, *T*/*T*_1_
*θ**: temperature ratio, *T*_2_/*T*_1_.
